# Impact of Fluorosis on the Tensile Bond Strength of Metal Brackets and the Prevalence of Enamel Microcracks

**DOI:** 10.1038/s41598-019-42325-4

**Published:** 2019-04-11

**Authors:** Giedrė Trakinienė, Gabrielė Petravičiūtė, Dalia Smailienė, Julija Narbutaitė, Justė Armalaitė, Kristina Lopatienė, Antanas Šidlauskas, Tomas Trakinis

**Affiliations:** 1 DDS, PhD, Lithuanian University of Health Sciences, Medical Academy, Department of Orthodontics, Kaunas, Lithuania; 2 DDS, Lithuanian University of Health Sciences, Medical Academy, Department of Orthodontics, Kaunas, Lithuania; 3 DDS, PhD, Prof., Lithuanian University of Health Sciences, Medical Academy, Department of Pediatric Dentistry, Kaunas, Lithuania; 4 Resident of orthodontics, Lithuanian University of Health Sciences, Medical Academy, Department of Orthodontics, Kaunas, Lithuania; 5 DDS, Department of Orthopedic Surgery, Respublican Hospital of Kaunas, Kaunas, Lithuania

## Abstract

The objective of this *in vitro* study was to determine the effects of dental fluorosis on the tensile bond strength of metal brackets bonded to human teeth and to evaluate the changes in the tooth enamel surface after debonding. The study sample consisted of 68 recently extracted human upper premolars, which were divided into 2 groups: the fluorosis group (34 fluorosed teeth) and the control group (34 healthy teeth). Identical premolar metal brackets were bonded on the buccal surfaces of the teeth. Both groups were submitted to a tension test using a universal testing machine. The number and length of the enamel microcracks in the buccal surface of each tooth were determined before bracket bonding and after debonding using a stereomicroscope. The percentage adhesive remnant index (PARI) was calculated after debonding. The results showed that the tensile bond strength (TBS) of brackets bonded to fluorosed teeth was 21.08% lower than that of brackets bonded to healthy teeth (p < 0.0001). The length of the enamel microcracks on fluorosed teeth increased by 47.4% after debonding, whereas the control group showed an increase of only 12.6%. The PARI showed lower values for fluorosed teeth in comparison to the control group (p = 0.047). In conclusion, dental fluorosis has a negative impact on tensile bond strength and the length of microcracks formed after bracket debonding.

## Introduction

One of the fundamental aims of orthodontic treatment with fixed appliances is to create an appropriate adhesive strength between the tooth enamel and the base of the bracket. Tensile bond strength (TBS) should be high enough to prevent debonding of brackets during treatment, and it should be low enough to minimize enamel damage during bracket removal^1,2^. If teeth have structural infractions, this factor may directly affect adhesion^3^.

One such infraction is dental fluorosis - a systematic noncariogenic enamel defect. Dental fluorosis is caused by the enamel’s exposure to excessive amounts of fluoride during the tooth developmental period^4^. The pathological changes in enamel are characterized by disorganized or abnormal crystal formation^5^. The surface of fluorotic enamel is extensively hypomineralized in the subsurface layer and well mineralized in the surface layer^6,7^.

Naturally or artificially added fluoride in drinking water is the main risk factor for dental fluorosis. Currently, approximately 380 million people are consuming artificially fluoridated water, and approximately 50 million people are drinking water with naturally occurring fluoride^8^. Currently, to achieve the best results in the prevention of dental caries, widespread use of fluoride toothpaste is evident. Fluoride-containing toothpaste, which is swallowed by young children, increases the risk of dental fluorosis^8,9^. The prevalence of enamel fluorosis is growing throughout the world, and the prevalence of orthodontic anomalies is not an exception in patients with this condition^10^.

Usually, evaluation of the fluorosis level is performed using the Thylstrup-Fejerskov classification (TF index). The TF index is based on visual clinical features of damaged teeth that coincide with changes in hydroxyapatite crystals. This index classifies dental fluorosis in terms of its absence (TF 0) through the presence of opaque lesions (TF 3) that blend to overtake the entire surface of the enamel, producing the appearance of white chalk (TF 4). In more advanced stages of fluorosis, there is a gradual loss of enamel and anatomical dental deformities (TF 5–9)^11^.

Furthermore, the adhesive strength between the enamel and a metal orthodontic bracket is not the only important factor that could be negatively affected by the structural changes of fluorotic teeth. Currently, orthodontic patients have high esthetic demands and pay more attention to possible enamel damage, which can manifest in the form of enamel microcracks (EMCs) after debonding^12,13^. In general, small cracks can be found in the enamel due to occlusal forces or temperature variations^14^. Usually, these small cracks do not lead to the fracture of the teeth, but over long periods, their growth may persist. This can cause deeper morphological changes of the tooth surface and create favorable conditions for bacterial accumulation, demineralization and further mechanical degradation, in addition to becoming a reason for teeth sensitivity^15^.

As previous studies have shown, more EMCs are apparent at the end of orthodontic treatment^16,17^. Zachrisson found a higher prevalence of cracks in debonded teeth compared to orthodontically untreated teeth^16^. Locally limited enamel surface cracks can provide stagnation areas with increased staining and plaque accumulation and therefore are at higher risk for the development of caries^16,17^. In severe cases, enamel fractures with flaking may occur, and the odds of these undesirable changes occurring in the enamel structure can be a few times higher than in orthodontically untreated teeth^18,19^. Cracks can act as precursors to the ultimate splitting of the entire tooth and can allow penetration into the dentin^20^. As fluorosed teeth already have structural changes, there might be a higher possibility for enamel damage during bracket removal^16^.

In addition, the removal of adhesive residue after bracket debonding with a dental bur may also lead to local enamel damage^21^. Thus, the amount of adhesive remnant after debonding is also an important factor^22^. Evaluation of the enamel surface after debonding of orthodontic accessories may be accomplished by means of the adhesive remnant index (ARI) recommended by Årtun and Bergland in 1984. The categories for scoring on a 4-point scale with respect to the bracket base are as follows: 0 = all adhesive left on the bracket base, 1 = more than half of the adhesive left on the bracket base, 2 = less than half of the adhesive left on the bracket base, and 3 = no adhesive left on the bracket base^23^. Other studies in the literature have developed a 5- or 6-point scale^24–27^.

However, previous studies did not reveal any analysis of the tensile bond strength of fluorosed teeth or changes in the enamel after bracket debonding; thus, the aim of this *in vitro* study was to determine the impact of dental fluorosis on the tensile bond strength of metal brackets bonded to fluorotic and nonfluorotic human teeth and to evaluate the changes in the enamel surface via the presence of microcracks after debonding.

## Materials and Methods

The present *in vitro* study was performed at the Department of Orthodontics and the Laboratory of Mechanical Engineering Faculty. The study was conducted in accordance with relevant guidelines and regulations. Bioethical approval was obtained from the University Bioethical Committee (No: BEC-OF-03). Written informed consent was obtained from all study participants.

The power analysis with G*Power (Version 3.1.9.2) statistical software was used to determine the sample size. The parameters adopted were as follows: significance level of 5%, power test of 80%, standard deviations of pilot samples of 2.6 MPa and 2.84 MPa, and smallest effect of interest of 2.

The sample size calculation was based on the following formula:

$$n=\frac{({{\rm{s}}}_{1}^{2}+{{\rm{s}}}_{2}^{2})\times {({{\rm{z}}}_{1-\frac{{\rm{\alpha }}}{2}}+{{\rm{z}}}_{1-{\rm{\beta }}})}^{2}}{{{\rm{\Delta }}}^{2}}$$

where n – the minimum sample size for each sample;

z_1-α/2_ = 1.96 and z_1-β_ = 0.84 if α = 0.05 and β = 0.2;

s_1,_ s_2_ - the standard deviations of pilot samples;

Δ - the smallest clinically important difference.

The sample size calculation showed that 29 specimens were needed in each group.

Teeth selection criteria were as follows: upper premolars recently extracted for orthodontic purposes with intact buccal enamel surface, no caries, no cracks from forceps, and no history of endodontic, prosthodontic or orthodontic treatment. The teeth were collected over a 2-month period and stored in distilled water according to the protocol of previous studies to avoid a significant influence of the storage medium on bond strength^27–30^. Teeth with a TF index score of 3 were assigned to the experimental group because these scores were the most prevalent in the population.

The extracted teeth were cleaned of soft tissue remnants and kept in the disinfectant solution *Korsolex Plus* (Bode Chemie GmbH) for 15 min. Later, they were washed for 1 min under a stream of water and blown dry. Subsequently, dental fluorosis was diagnosed according to the Thylstrup-Fejerskov Index (TF)^11^. One of the authors of this study (JN) has additional training in fluorosis diagnostics and carried out the tooth examinations. Photos of various fluorosis severity stages were on-hand during the examination. Specimens were stored in an isotonic solution at room temperature during the experiment to prevent dehydration. The solution was changed daily to avoid bacterial growth.

Out of 305 collected teeth, 68 met the inclusion criteria and were used for the study: 34 fluorosed teeth (experimental group) and 34 healthy teeth (control group).

All the teeth we embedded in the same shape of iron rings filled with class IV gypsum to ensure stability of the samples The teeth were fixed so that the buccal surfaces were parallel to the force modeling direction of the universal testing machine. Before the bonding of the bracket, the buccal surface of each tooth was polished with a rubber cup and nonfluoridated pumice, rinsed with water and dried with compressed air for 20 seconds. Then, the prepared enamel area was etched with 37% phosphoric acid (Gel ETCH, 3 M Unitek Germany) for 30 seconds, washed and dried with oil-free compressed air until a white frosty appearance was observed. The etched enamel surface was conditioned with TruLock bond (Rocky Mountain Orthodontics) and light-cured for 15 seconds (Translux Wave, Heraeus Kulzer, Germany, 1000 mW/cm^2^). Sixty-eight identical Roth prescription 0.022 slot metal upper first premolar brackets (Discovery Dentaurum Germany) were bonded 2 mm gingivally to the buccal cusp tip in the center of the clinical crown with TruLock light cured adhesive resin (Rocky Mountain Orthodontics). Each bracket was positioned using a 100 g weight – 9.8 N force through the adapter to the buccal tooth surface by the same individual to ensure the standardization of the thickness of the luting agent. A dental probe was used to remove any residual adhesive around the brackets. The luting agent was light-cured for 30 seconds. All the specimens were stored in containers with isotonic solution for 1 hour until the resin was completely polymerized, and then, the samples were used for the tensile bond strength test^27^. After bracket debonding, each sample was analyzed by stereomicroscopy for the calculation of PARI. Next, the residual adhesive on each tooth was removed using a 12-bladed carbide bur on a low-speed contra-angle handpiece (less than 20 000 rpm) without water cooling and was followed by polishing with a rubber cup. The bur was changed after every 10 teeth in every group. Subsequently, stereomicroscopic reevaluation of EMCs was performed.

**Figure 1:**
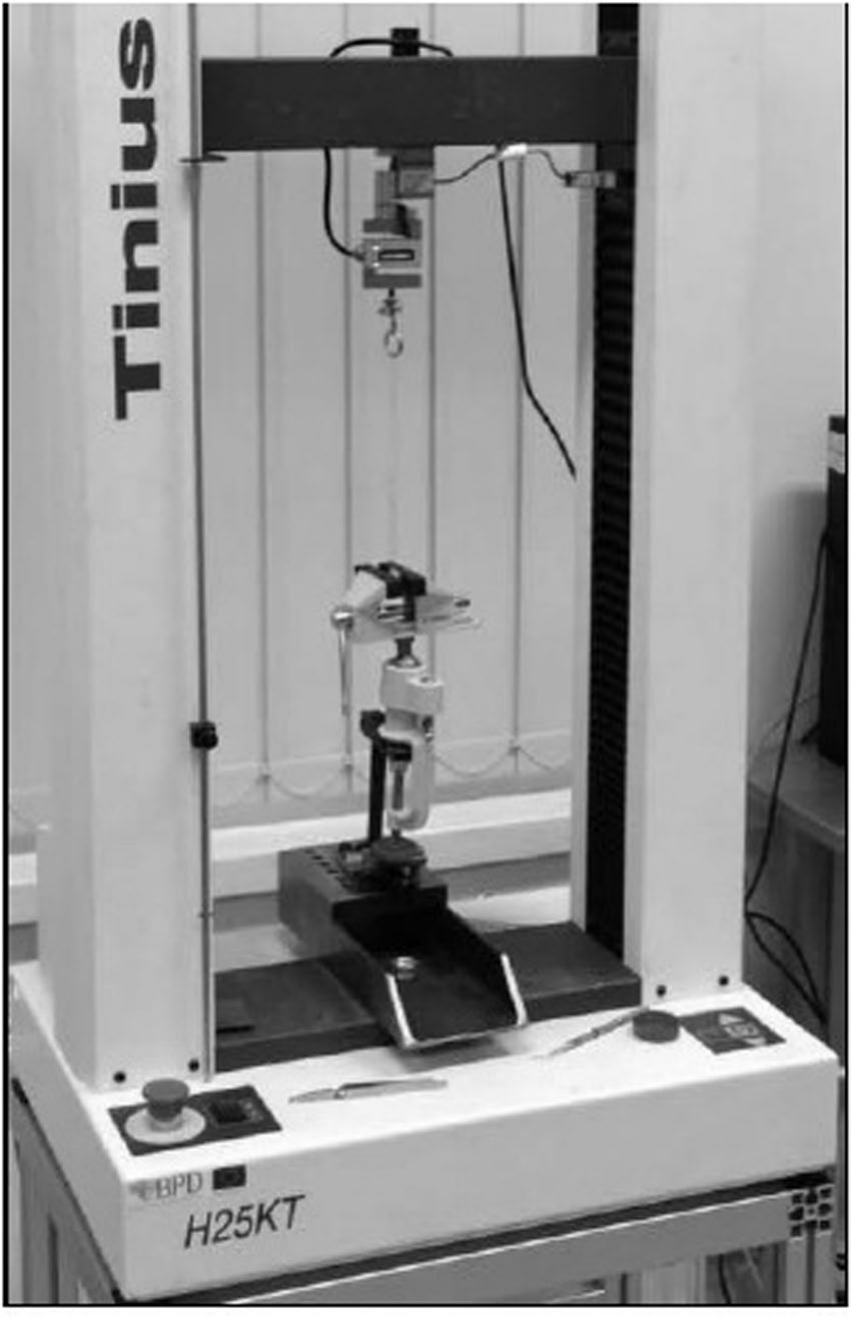
Universal testing machine (Tinius Olsen H25 KT) for the measurement of tensile bond strength^27^.

The tensile bond strength (TBS) was measured in the Department of Mechanical Engineering at the University. The standard “U” shape loops were bent from 0.36 mm diameter orthodontic archwire (Remanium No. 751-001-00, Dentaurum. Germany) and adjusted to the universal mechanical testing machine (H24KT, Tinius Olsen, England). These loops were fixed to the brackets with ligatures. The testing machine was used at a crosshead speed of 1 mm/min until the bracket was debonded from the tooth (Fig. 1). The highest debonding forces (N) of the brackets were recorded automatically by a digital software measurement system. The system consisted of a force sensor (SS50, Wagner Instruments, USA, 250 N × 0.1 N) and a controller with a display (BGI, Wagner Instruments, USA). TBS was counted using the force’s value and the base of the bracket area value (1 MPa = 1 N/mm^2^).

Analysis of the number and length of the enamel microcracks (EMCs) before bracket placement and after its removal was performed using a digital microscope camera (ZEISS, AxioCam Mrc5) and a 20x magnification by the stereomicroscope (ZEISS, AxioZoom). For the achievement of the same magnification, the distance from the buccal surface of the tooth to the stereomicroscope lens was standardized. Using the AxioVision software program, the digital zoom scale was fixed in the photos. EMCs are not visible when their direction coincides with the direction of a light source^26^. For this reason, each sample was rotated 360° around the tangent, perpendicular to the buccal enamel surface, and the source of light was always positioned at 45° to the buccal surface. The length of EMCs was measured in the photos using a fixed scale.

The adhesive remnant index (ARI) was evaluated in the stereomicroscopic digital photos using the *Sketchandcalc* software program. The percentage adhesive remnant index (PARI) on the tooth surface was calculated using the following formula:

$${\rm{PARI}}=100 \% \,-\,{\rm{B}} \% $$

PARI – percentage adhesive remnant index on the tooth surface

B – residual adhesive remnant on the bracket base

The values were divided into 6 groups according to the percentage of adhesive remnant on the tooth surface^24^.

### Statistical analysis

Statistical analysis was performed using the IBM SPSS Statistics 22.0. Descriptive statistics were reported as the mean and standard deviation (SD). Hypotheses of interrelations between characteristics were verified using Pearson chi-squared test (*X*^2^), Tukey post hoc test and one-way ANOVA. P-values less than 0.05 were considered significant. The power of the analysis was 0.8. Intraexaminer reliability was assessed by re-evaluating the number and length of EMCs and the reliability of PARI scoring two weeks apart for all teeth. Cohen’s Kappa coefficient showed very good intraexaminer reliability for EMCs (0.901) and for PARI (0.920).

## Results

The study sample of the present study consisted of 68 specimens: 34 dental units in the fluorosed teeth group and 34 dental units in the intact teeth group.

### Comparison of tensile bond strength (TBS)

There was a statistically significant difference in TBS between the experimental and control groups (p < 0.0001). The mean TBS of fluorosed enamel was 21.08% lower than the mean TBS of healthy teeth (Table 1).

**Table 1:** Comparative data of brackets tensile bond strength. Max indicates maximum; Min indicates minimum; SD - standard deviation.

Group	Tensile bond strength (MPa)				
	Mean	SD	Min	Max	P value
Fluorosed teeth (n = 34)	10.14	2.6	6.65	14.38	<*0*.*0001*
Intact teeth (n = 34)	12.85	2.84	8.21	17.91	

### Evaluation of enamel microcracks (EMCs) before bracket placement and after debonding

There were no statistically significant differences between the groups comparing the mean overall number (p = 0.128) and length (p = 0.441) of EMCs before bracket bonding, although the mean values were higher in the control group than in the fluorosed group (Table 2).

**Table 2:** Comparative data of enamel microcracks (EMCs) number and length before bonding and after removal of brackets (SD - standart deviation; *statistically significant).

Group	Number of EMCs			Length of EMCs (μm)		
	Before bonding (Mean ± SD)	After removal (Mean ± SD)	P	Before bonding (Mean ± SD)	After removal (Mean ± SD)	P
Fluorosed teeth (n = 34)	1.26 ± 1.42	1.50 ± 1.44	0.46	3.56 ± 3.04	5.25 ± 3.54	0.007*
Intact teeth (n = 34)	1.41 ± 1.16	1.76 ± 1.18	0.218	4.04 ± 2.42	4.55 ± 2.52	0.265

Evaluation of the number of EMCs after debonding indicated that 19% of fluorotic teeth showed new EMC appearance compared to 24.8% of intact teeth with new EMCs; however, the difference was not statistically significant (p = 0.111). Pearson analysis showed no correlation between TBS values and changes in the number of EMCs in either group (p = 0.668. r = 0.15).

After further examination, a significant change in EMC length after bracket removal in the fluorosed teeth group was found (p = 0.007). In this group, the length of EMCs increased by 47.4%, while in the control group, it increased only 12.6% after bracket removal (Table 2). In addition, a correlation between TBS values and changes in the length of EMCs was found (p = 0.044. r = 0.58); thus, increasing TBS induced an increase in the length of EMCs in the fluorosed teeth group, while in the controls, these changes were very small.

### Percentage adhesive remnant index (PARI) analysis

**Figure 2:**
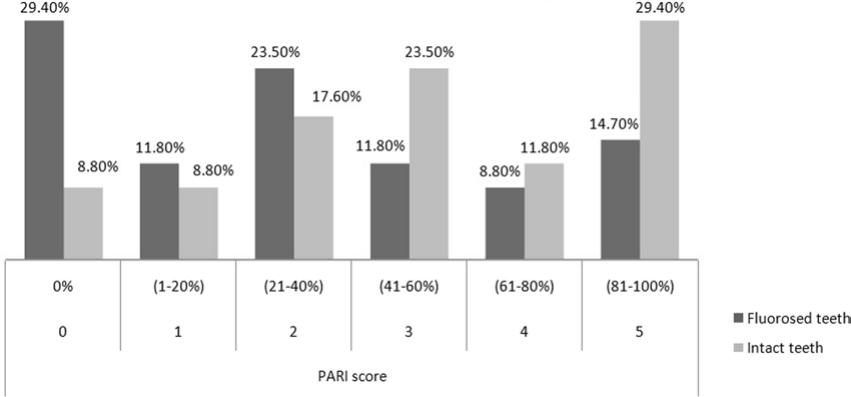
Distribution of percentage adhesive remnant index (PARI) score in experimental and control groups.

In the experimental group, the amount of adhesive remnant on the teeth was significantly smaller than that in the control group (p = 0.014) (Fig. 2). Pearson analysis showed a positive correlation between PARI values and TBS (p = 0.045. r = 0.55); thus, the increase in TBS caused an increase in adhesive remnant score.

## Discussion

A recent study was conducted to evaluate whether dental fluorosis had an effect on the tensile bond strength. One aspect of the discussion was the minimum value of the tensile bond strength. Reynolds suggested that the minimum clinically acceptable tensile bond strength was 5.9–7.8 MPa^31–33^. In the present research, fluorosed enamel showed significantly decreased TBS. Despite this observation, the decreased TBS values in the fluorosed enamel group exceeded the minimum recommended TBS. However, *in vitro* studies usually show bond strength values up to 40% higher than are observed in comparable *in vivo* situations^34,35^. In a recent study, when the *in vitro* tensile bond strength was reduced by 40%, the mean bond strength and minimum bond strength assessed for fluorosed teeth barely exceeded 6 MPa and 4 MPa, respectively. Thus, the bond strength of many fluorosed teeth may not be high enough for effective orthodontic treatment, and additional enamel preparation may be useful for improving the bond strength.

Previous studies on fluorotic teeth revealed that in the outer, hypermineralized fluorotic enamel layer, hydroxyapatite crystals are changed by fluorapatite crystals. This is the reason why such tooth surfaces become more resistant to acid^9,36,37^. Silva-Benitez *et al*. recommended prolonging enamel etching time in moderate fluorosis cases, but in severe cases, she advised combining air abrasion and enamel etching procedures^38^. However, due to the histoanatomic differences of the fluorosed teeth, it is complicated to predict a suitable etching time^1^. In contrast, studies by Najafi *et al*. and Mendes *et al*. found that prolonged enamel etching time combined with air abrasion or enamel preparation by laser did not increase the TBS of the brackets^39,40^.

There is a third opinion in the literature that no additional preparation of fluorosed enamel is required before bracket placement because the TBS values of the fluorosed teeth do not change^32^. These disparities could be because different studies used teeth with unequal degrees of fluorosis. Furthermore, a general recommendation is that teeth should be used for experimentation within 1 month after harvesting^31^. In the present study, the protocol for tooth selection and enamel preparation was standardized: the TF index value was 3 and the enamel in both groups was etched for the same amount of time (30 seconds).

Another question evaluated in the present study was whether dental fluorosis was related to a higher possibility for enamel damage during bracket removal. In this study, the effect of bracket debonding on the enamel structure was evaluated in terms of EMC characteristics, including the length and number of EMCs before bracket placement and after their removal. In fluorosed teeth, a significant change in EMC length, but not in number, was found. The lengthening of enamel microcracks can be explained by adhesive resin infiltration through the cracks to the hypomineralized enamel subsurface layer, and in this way, the EMCs have the opportunity to spread during debonding^9^. Moreover, the thick, undamaged, hypermineralized outer enamel layer gives protection against the formation of new microcracks^41^. Dumbryte *et al*. stated that the existence of EMCs and certain characteristics, such as their location and visibility before bracket placement, were closely linked to major tooth structure defects after debonding. The possibility of irreversible enamel defect formation increased in 20.4% of cases^42^. The results of the present study showed that in fluorosed teeth, EMC length changes were as high as 47.4%, and the increase in TBS positively correlated with the increase in EMC length in the fluorosed teeth group.

The relevant issue of debate is the amount of adhesive remnant on the tooth surface after bracket removal. According to some authors, a minimal amount of ARI on the tooth surface is desirable since it helps to avoid additional removal procedures that can be iatrogenic to enamel^42^. In contrast, Xiao-Chuan Fan suggested that the loss of the connection in the bracket–adhesive resin intersection during debonding is a safer procedure because it protects tooth enamel from direct damage during bracket removal^43^. In the current study, a modified PARI index showed that the adhesive remnant on the tooth surface after bracket debonding was directly associated with the TBS of brackets; the increased bond strength caused an increase in the value of the ARI index. The amount of adhesive remnant on fluorosed teeth was statistically lower than that on healthy teeth, and the lengthening of EMCs was higher. These results confirmed that TBS on fluorosed teeth was lower and that the enamel was more fragile than that in healthy teeth.

### Limitations of the study

Since one of the purposes of this investigation was to evaluate, in detail, qualitative EMC characteristics, an *in vitro* study model was chosen. It allowed the authors to collect the proper sample size and to analyze and make direct, precise measurements of the length and number of EMCs. However, it is important to emphasize that *in vitro* bond strength is higher than that *in vivo* (because of the oral humidity, etc.). Thus, the increase in EMCs may be greater in the present study than in a clinical situation^25^. Therefore, conducting *in vivo* studies is recommended to confirm the presented results and to address limitations of the stereomicroscope for evaluating the exact dimensions of enamel microcracks.

## Conclusions

Dental fluorosis has a negative impact on tensile bond strength and the length of microcracks formed after bracket debonding.
